# Age-Specific Incidence of A/H1N1 2009 Influenza Infection in England from Sequential Antibody Prevalence Data Using Likelihood-Based Estimation

**DOI:** 10.1371/journal.pone.0017074

**Published:** 2011-02-23

**Authors:** Marc Baguelin, Katja Hoschler, Elaine Stanford, Pauline Waight, Pia Hardelid, Nick Andrews, Elizabeth Miller

**Affiliations:** 1 Immunisation, Hepatitis and Blood Safety Department, Health Protection Agency, London, United Kingdom; 2 Centre for the Mathematical Modelling of Infectious Diseases, London School of Hygiene and Tropical Medicine, London, United Kingdom; 3 Respiratory Virus Unit, Virus Reference Department, Health Protection Agency, London, United Kingdom; 4 Vaccine Evaluation/Seroepidemiology Unit, Health Protection Agency, Manchester, United Kingdom; 5 Statistics Unit, Health Protection Agency, London, United Kingdom; National Institutes of Health, United States of America

## Abstract

Estimating the age-specific incidence of an emerging pathogen is essential for understanding its severity and transmission dynamics. This paper describes a statistical method that uses likelihoods to estimate incidence from sequential serological data. The method requires information on seroconversion intervals and allows integration of information on the temporal distribution of cases from clinical surveillance. Among a family of candidate incidences, a likelihood function is derived by reconstructing the change in seroprevalence from seroconversion following infection and comparing it with the observed sequence of positivity among the samples. This method is applied to derive the cumulative and weekly incidence of A/H1N1 pandemic influenza in England during the second wave using sera taken between September 2009 and February 2010 in four age groups (1–4, 5–14, 15–24, 25–44 years). The highest cumulative incidence was in 5–14 year olds (59%, 95% credible interval (CI): 52%, 68%) followed by 1–4 year olds (49%, 95% CI: 38%, 61%), rates 20 and 40 times higher respectively than estimated from clinical surveillance. The method provides a more accurate and continuous measure of incidence than achieved by comparing prevalence in samples grouped by time period.

## Introduction

Rapid understanding of the severity profile and transmission dynamics of an emerging pathogen is essential in order to anticipate demands on health care resources and develop appropriate public health interventions. While cases reported through clinical surveillance systems, such as statutory notifications or sentinel physician reporting schemes, may reflect the time course of the wave of infection, they may not provide an accurate measure of the infection rate in the population. For A/H1N1 (2009), the occurrence of asymptomatic infections or clinical cases for which no medical care is sought can lead to gross underestimation of the true incidence of infection [1], [2] and overestimation of severity [3], when using clinical surveillance data.

We previously reported the results of a rapid serosurvey that provided early estimates of the age-specific incidence of infection with the A/H1N1 (2009) virus during the first wave in England [2]. For this, age-specific incidence estimates were derived by testing sequential serum samples grouped by calendar month and comparing the prevalence of antibody to the H1N1 (2009) influenza virus in that month with that in the same age-group in baseline samples collected prior to its arrival in the United Kingdom. This method has a number of limitations. First, the monthly samples are distributed over a 30 day period during which incidence may be changing, particularly at the height of the wave. Second, the variable time to seroconversion across individuals means that even if all samples were taken on the same date they reflect incidence at different times in the previous weeks. Third, derivation of incidence by comparing prevalence between time points reduces the precision of the estimate for a given sample size and may result in negative point estimates for groups in whom incidence is low [2], unless a method is used that prohibits such estimates.

We report the age-specific incidence of infection in the second wave in England using a novel statistical method for analyzing sequential serology data. In this example, we utilize information on the temporal distribution of cases as estimated from clinical surveillance data combined with information on seroconversion interval and the exact timing of each serum sample to estimate incidence by week. Our method provides a better definition of the age-specific incidence of an emerging pathogen over time- information that is critical for the parameterization and validation of predictive models and for assessing disease severity.

## Materials and Methods

### Serologic data

Serum samples were residual aliquots obtained from chemical pathology and microbiology laboratories in eight regions in England and sent to the Manchester Seroepidemiology Unit (SEU), after being irreversibly unlinked from any patient identifying information according to the standard SEU protocol [4]. Due to limitations on the numbers of samples that could be tested each month, continuous collection of sera throughout the second wave was only carried out for those aged under 45 years [5]. The analysis was therefore restricted to 2684 samples from individuals aged 1 to 44 years taken from 1^st^ September 2009 to 23^rd^ February 2010. The information available for each sample was date of collection, age, sex and collecting laboratory. Sera were tested for antibody to H1N1 (2009) influenza virus by haemagglutination inhibition (HI) using standard methods as previously described [2], [5] at the Respiratory Virus Unit at the Centre for Infections Health Protection Agency, United Kingdom. The starting dilution for the HI assay was 1∶8 and sera were titrated by doubling dilution to determine absolute end point titers ending at a dilution of 1∶16384. As used previously [2], the threshold titre for positivity used in the analysis was ≥32, four times the minimum level of detection. Comparison of the HI titer distribution in pre-pandemic and post- pandemic sera from children under 15 years of age showed this threshold to be a highly specific marker of recent infection [5].

### Ethical review

Approval for the unlinked anonymous testing of sera from the SEU collection was obtained from the National Research Ethics Service (NRES reference number 05/Q0505/45).

### Time course of the second wave

Information on the distribution of clinical cases of H1N1 (2009) by age group and week in England between August 2009 and February 2010 was obtained from the weekly estimates of the number of clinical cases made by the Health Protection Agency (HPA) during the course of the pandemic, as previously described [6]. Briefly, these estimates used health care consultation rates by age for influenza-like-illness (ILI) adjusted for the estimated proportion of patients with ILI seeking health care, and the proportion consulting in whom H1N1 (2009) infection was laboratory-confirmed by a validated PCR assay [7].

### Distribution of seroconversion interval

For estimation of the distribution of seroconversion intervals (here defined as the time taken to reach an HI titer of ≥32) in days since symptom onset, serum samples from named patients with suspected pandemic influenza sent to the Respiratory Virus Unit by clinicians for HI antibody testing as part of their clinical work up were cross checked against the database of individuals with PCR confirmed infection held by the Respiratory Virus Unit. This provided a data set of individuals with serum samples taken at various intervals after onset of a confirmed infection. The date of onset of symptoms was obtained by follow up via the patient's general practitioner if not supplied with the serum sample or available via the national database established by the HPA early in the pandemic to facilitate tracking of confirmed cases. This linkage yielded 115 HI titers from patients with known dates of sample and symptom onset for analysis.

Not all infected individuals attain a titer of ≥32 by HI [2], [5]. We assume that the distribution *A(t)* of the seroconversion interval *t*, following infection, is given by a mixture distribution where 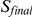 is the probability of ever seroconverting conditional on infection, and conditional on seroconversion, the time from infection to seroconversion is Weibull. We thus need to evaluate three parameters to evaluate the interval to seroconversion distribution, 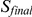 and the two parameters from the Weibull distribution.

Parameters for the Weibull function describing the time from infection to seroconversion were sampled using Markov Chain Monte Carlo (MCMC) and a simple Metropolis-Hasting algorithm with uniform priors for the three parameters. To assess convergence, several chains with different starting values were run in parallel. Iterations were stopped when enough samples were generated to see agreement between the estimates from the different chains [8]. We assumed that seroconversion interval and proportion seroconverting did not differ by age group [5].

### Derivation of the incidence

We start, for an age group *i*, with a family of possible incidence curves 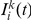 parameterized by *k*. The aim of the method is to assess from the sequence of serologic samples which incidence curves are the most likely to have generated the observed sequence of seropositivity in the samples by working out their posterior distribution. We assume in this paper that the incidence is constructed from the proportion of cases by week over time based on the normalized HPA clinical case estimates multiplied by an unknown cumulative incidence. We thus restrict the estimation of the incidence curves for the different age groups to one parameter, the cumulative incidence i.e. the final proportion in the age group that was infected during the second wave. The final result of the calculation is then a posterior distribution of cumulative incidences based on a likelihood function given by the sequence of positivity observed in the samples and a prior distribution for the cumulative incidences. We assume these priors to be uniform over [0,1].

To estimate when the people developing symptoms will achieve an HI titer of ≥32), the distribution *A(t)* of intervals from onset of symptoms to seroconversion is needed. Knowing this distribution, the number of people who seroconvert at time *s* after onset of symptoms (or at an equivalent time after exposure if they are asymptomatic) is given by 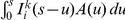; summing all the people who had onset of symptoms at time *s-u* weighted by the proportion of them who seroconvert in an interval of time *u*. This assumes that all people who seroconvert through infection are initially seronegative (HI titer <32). The change of seroprevalence 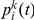 of antibody in age group *i* is then given by summing over the course of the epidemic (starting at *t = 0*) all the individuals who seroconverted and adding them to the pre-epidemic baseline 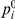: 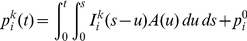
(1)


Then, assuming that samples are drawn at random from the population for whom we wish to derive incidence, i.e. the population of England, and that there is no spatial heterogeneity, the probability of having a positive sample at time *t* is given by 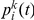.

Assuming independence of the samples, the likelihood of observing the data *D* given the incidence 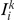 is given by:
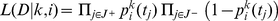
(2)with 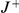 denoting the set of positive samples, 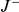 the set of negative samples and *t_j_* the time of collection of sample *j*. Following Bayes' theorem, the probability of incidence 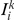 given the observed data is proportional to 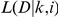 times the prior probability of the incidence (we assume 0-1 uniform priors for the rest of the study).

### Algorithm to compute incidences incorporating uncertainty

To draw a sample from the posterior distribution of the cumulative incidences (in each age group, so *i* is fixed) while taking into account the uncertainty from the interval to seroconversion, we used a rejection sampling algorithm. The algorithm will lead to the correct distribution as soon as the acceptance rate is proportional to the likelihood function [9]. We thus employed the following algorithm:

1. Generate a sample 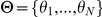 of distributions of the parameters for the interval to seroconversion distribution using MCMC.
2. Draw a value from 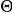 and from the prior of the cumulative incidence (uniform over [0,1])
3. Accept the sample with a probability 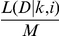, with *M* a constant such that 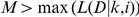

4. Repeat until the required number of samples is achieved.

Given that the evaluation of our likelihood function only depends on one parameter, it is straightforward to find a constant *M* such that 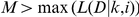. For this, we have computed the likelihood for a grid of values of cumulative incidences and samples of 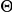 and have taken *M* as twice the maximum value found. The bigger the value of *M*, the lower the probability of acceptance will be, but the smaller the value of *M* the bigger the risk of numerical inaccuracies. The value of the likelihood function has been checked during the analysis to be below the chosen value of *M*. More sophisticated approaches could be taken to choose a more optimal value of *M* though the value chosen demonstrated computational efficiency for our purpose.

We apply this method to find the cumulative incidence in the age-groups 1–4, 5–14, 15–24 and 25–44 years during the second wave of H1N1 (2009) in England using the distribution of cases by week from clinical surveillance data to describe the temporal variation of the incidence. The seroprevalence is reconstructed by integrating Equation 1, with a time step of one day, the weekly cases being spread uniformly over the week. The candidate final cumulative incidences are assigned a likelihood function depending on the sequence of positivity of the samples in each age group by using Equation 2. As the prior distributions of the cumulative incidences are uniform over [0,1], to find the posterior distribution of the incidences, the likelihood function is renormalized so that it sums to one. The baseline seroprevalence was defined as the proportion positive among the 1272 samples taken during the last two weeks of the first wave and the first two weeks of the second wave (August 17^th^ to September 13^th^ 2009).There were significant differences in prevalence of antibody to H1N1(2009) virus between regions in the samples collected in September and October 2009 (consistent with the different attack rates between regions in the first wave [2]). However, these regional differences were considerably reduced by February 2010 [5] and for this methodological example data have been combined across regions for all time points.

Estimation of total number of infections and comparison with estimates derived from clinical surveillance.

For estimation of the total number of infections by age in the second wave, the final cumulative incidence was multiplied by the number of individuals in that age group in England, as obtained from the Office of National Statistics Mid 2008 population [10]. These numbers were then compared with the cumulative number of clinical cases in each age group as estimated by the HPA during the second wave [6].

## Results

**Figure 1:**
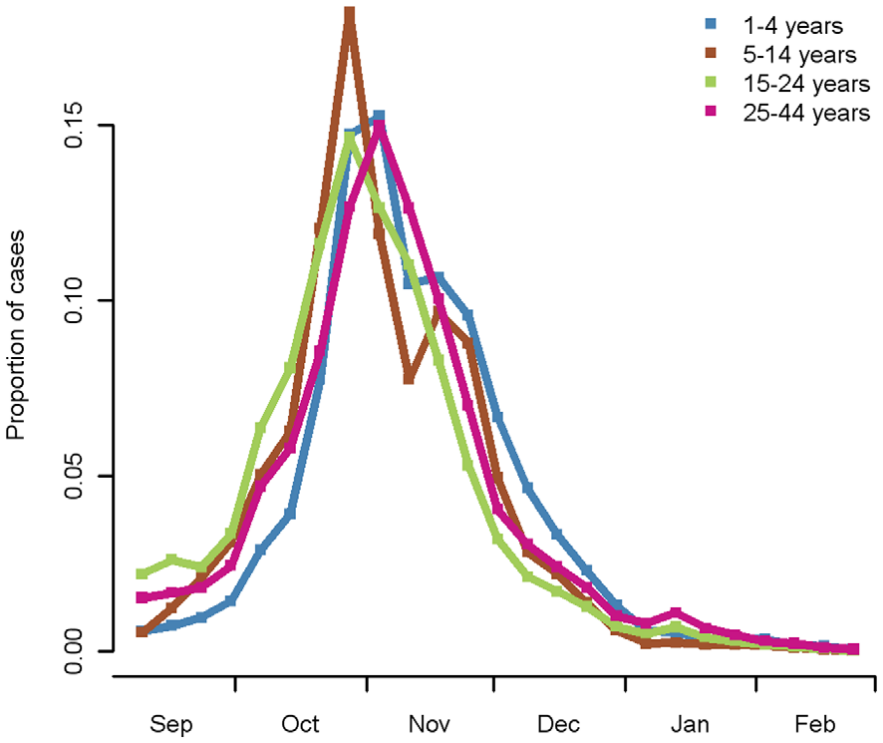
Proportion of clinial cases by week. Proportion of clinical cases by week for the second wave for four age-groups (1–4, 5–14, 15–24, 25–44 years) derived from clinical surveillance data.

The distribution of clinical cases in the second wave by week was similar in the four age groups, though with an earlier peak in the 5–14 and 15–24 year olds (Figure 1). The drop in clinical incidence in the 5–14 year olds in November coincided with school closure for half term.

### Distribution of time to seroconversion

**Figure 2:**
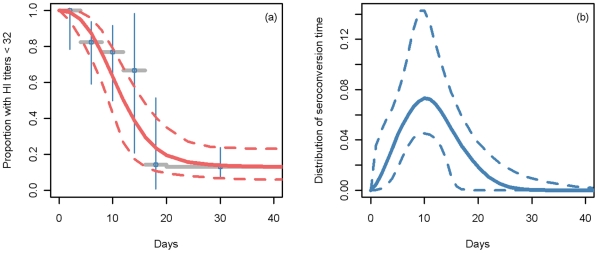
Estimation of the interval to seroconversion. a) Proportion of individuals with HI titer <32 by interval since symptom onset: blue lines and points show the proportion in four-day intervals with confidence intervals and the red curve show the fitted parametric inverse cumulative distributions with the 95% CI (credible intervals) and b) distribution of the time to seroconversion since symptoms with 95% CI.

The posterior mean values for the scaled Weibull distribution parameters are 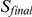 = 0.87 [0.77, 0.94], shape = 2.42 [1.44, 4.26] and scale = 12.87 [9.55, 16.98]. This means that, among those who do seroconvert (87%), 50% will have seroconverted by the 12^th^ day and 95% by the 21^st^ day (Figure 2). The posterior covariance matrix for the parameters (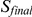, shape and scale) is:
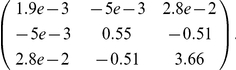



### Estimated cumulative incidence in the second wave

**Figure 3:**
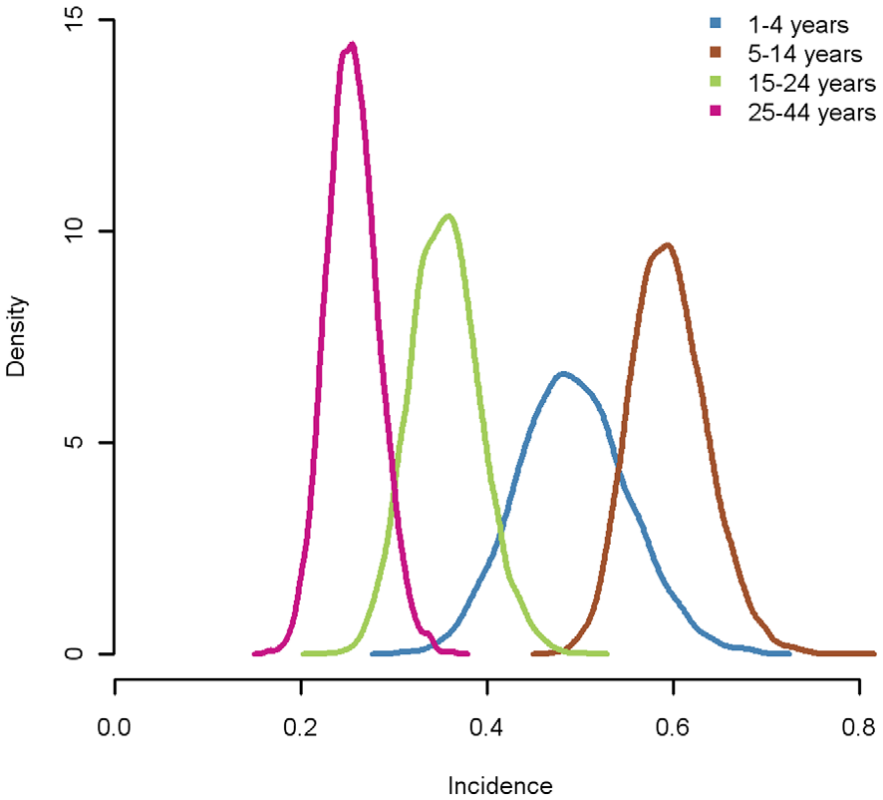
Posterior distribution of the cumulated incidences. Estimated cumulated incidence distributions for age-groups 1–4, 5–14, 15–24, 25–44 years during the second wave.

Posterior distributions for the estimated cumulative incidence are shown in Figure 3 for the four age groups in the analysis. The accuracy of the estimate depends on the number of samples. Even in the 1–4 year age group where there is a limited number of samples (n = 274 over a 6 month period, Table 1), the distribution is relatively well localized. The estimated cumulative incidence is highest among 5–14 year olds with around 59% of individuals in this age group, representing some 3.5 million children, likely to have been infected in England in the second wave (Table 1). Children aged 1 to 4 years have the second highest cumulative incidence (49%) with 1.21 million likely infections. In the older age groups, cumulative incidence is still high (35% and 25% in the 15–24 and 25–44 year age groups respectively resulting in an estimated 2.4 and 3.7 million infections respectively. When compared with the number of clinical cases estimated by the HPA method in the second wave [6], the estimated number of infections were between 43 and 21 fold higher depending on the age group (Table 1).

**Table 1:** Baseline percentage of samples with HI titer ≥32 before the start of the second wave, number of samples tested and estimated cumulative incidence (with 95% credible intervals) over the second wave of H1N1 (2009) infection in England starting from September 1^st^ 2009 according to age group.

Age group and number of samples (n) tested September 2009 to February 2010	Prevalence of HI titers≥32 before the 2^nd^ wave	Estimated cumulative incidence over the 2^nd^ wave		Population size (2008) in thousands	Estimated infections in thousands from serology		Estimated clinical cases in thousands from surveillance data **	Number of infections per estimated clinical case
		mean	95% CI*		mean	95% CI		
1–4 years, n = 274	9.8%	49	38, 61	2,462	1,210	935; 1,510	28	43
5–14 years, n = 840	14%	59	52, 68	5,904	3,500	3,080; 4,030	170	21
15–24 years, n = 604	15.3%	35	28, 43	6,862	2,430	1,950; 2,980	102	24
25–44 years, n = 966	12.3%	25	20, 31	14,417	3,670	2,930; 4,510	133	28

*CI, credible intervals,

**as described in reference 6.

### Change in seroprevalence and seroincidence during the second wave

**Figure 4:**
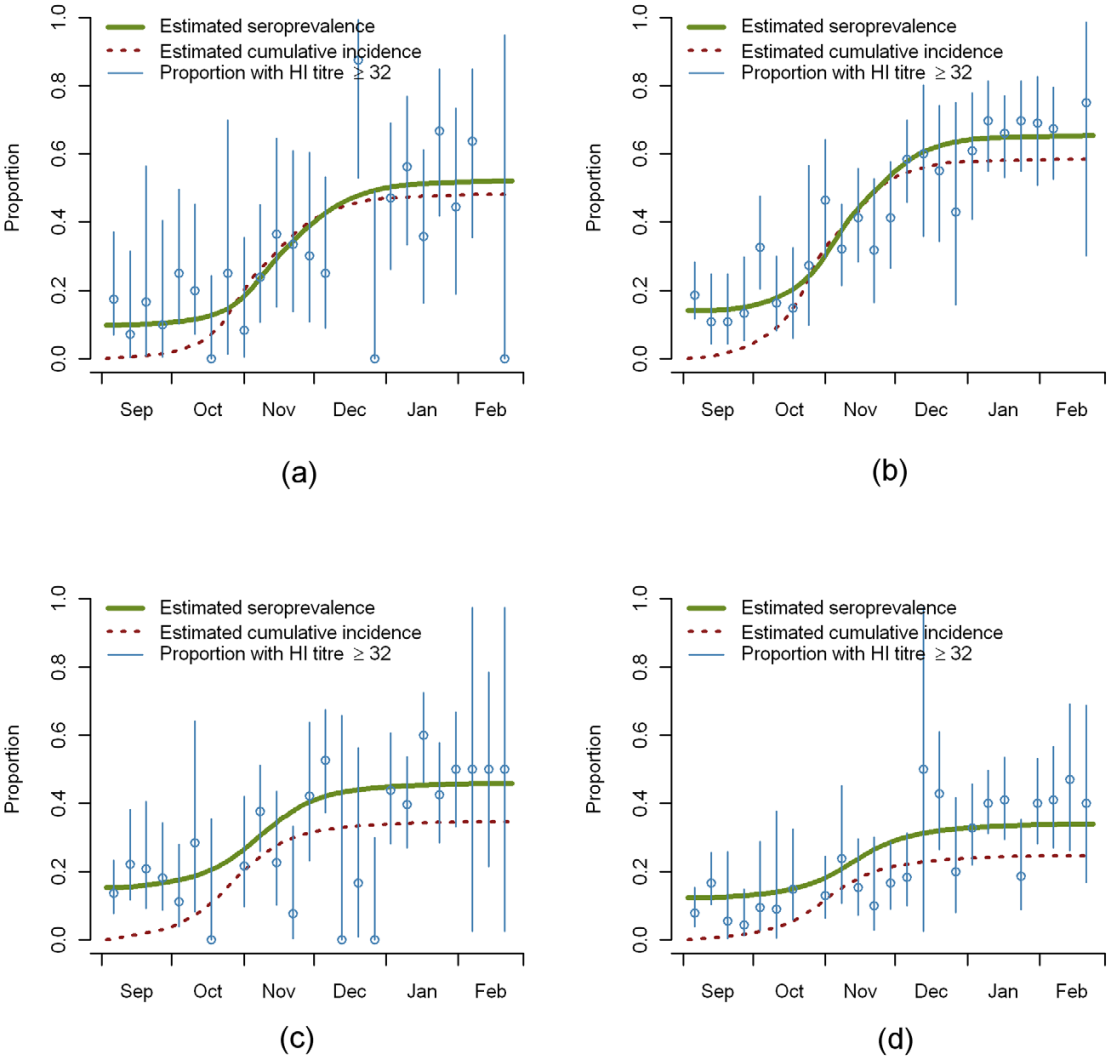
Changes in seroprevalence and cumulative incidence over time. Estimated changes in seroprevalence and cumulative incidence compared with proportion with HI titer ≥32 by week by age group a) 1–4 years, b) 5–14 years c) 15–24 years d) 25–44 years.

For each age-group, the observed proportions (with 95% CI) of samples with HI titers ≥32 when grouped by week are shown in Figures 4a–d. Also shown is the estimated cumulative seroprevalence and estimated cumulative seroincidence by day, each starting from the baseline at the beginning of the second wave. In contrast to the observed weekly seroprevalences, the estimated cumulative seroprevalence increases monotonically.

## Discussion

A key element of the contingency planning for an influenza pandemic in the United Kingdom has been the development and validation of real time transmission models that can predict the future impact on health care resources and evaluate the optimal deployment of interventions such as school closure and vaccination [1], [11], [12]. Accurate and timely measures of the age-specific incidence of infection as the pandemic evolves are essential for model development and parameterization and are ideally obtained from sequential serologic surveys. Estimates of the number of infections are also essential for assessing severity as fatality and hospitalization rates may be overestimated if denominators are based on cases accessing health care [3], [13]. The method described here facilitates the generation of incidence data by the novel application of a likelihood-based estimation to the analysis of sequential serologic data. A similar likelihood approach has been used recently for the estimation of the final attack rates but without using surveillance data to obtain a continuous incidence curve necessary for example to do modeling in real time [14].

The use of serologic data to estimate incidence of infection requires an antibody assay that is a sensitive and specific marker of the immune response to recent infection. The HI test developed by the HPA had high specificity [5] and as no other influenza viruses were circulating during the second wave in England the issue of the development of cross reactive antibodies,which may occur with exposure to antigenically-related influenza viruses, did not arise. The HPA HI test has been extensively used to measure antibodies induced by H1N1 (2009) vaccines [15], [16] and detected antibodies at a titer of ≥32 in 88% of individuals with confirmed infection tested more than 21 days after symptom onset. Some of the samples with a low HI titer had antibodies to the H1N1 (2009) virus detected by micro-neutralization assay, consistent with an immune response to recent infection. The micro-neutralization is a more sensitive assay than HI as it measures antibodies to other neutralizing epitopes in addition to the haemagglutinin antigen. However, it is a more time consuming assay to perform and would not be suitable for the rapid generation of serologic data for estimation of sero-incidence.

We assumed that HI antibodies developed in response to infection remain at a titer of ≥32 for at least 7 months such that individuals infected in August 2009 would still be seropositive if tested in February 2010. Given that this threshold is a correlate of protection it seems reasonable to assume that HI titers would be maintained at this level in individuals with naturally-acquired immunity. Generation of data on the kinetics of decay of HI antibodies would ideally require sequential samples from a cohort with known date of infection and followed up for antibody persistence. In our seroconversion data set there was no evidence of a decline in titers with time since onset after the seroconversion peak at 10 days though we had few individuals with samples taken >3 months after infection. However, if our method was applied to estimate cumulative incidence over a more extended period, for example from a baseline at the start of the first wave in April 2009, and if titers were shown to decline below the HI threshold within this time, a sero-reversion factor could be incorporated using the knowledge of the kinetics of the antibody decay curve.

In applying the method to the serologic data from the second wave, we assumed the incidence to be zero at baseline and that the baseline prevalence was accurately known. The likelihood estimation method though can still be applied if the baseline seroprevalence at the start of a pandemic wave is unknown. Providing that there are sufficient samples taken through the course of the pandemic, the baseline could be estimated as part of the parameter estimation. In this instance, the likelihood function measures the chance of observing the data given the incidence curve derived from clinical surveillance data, starting from the candidate pandemic baseline. By adding a new quantity to estimate the uncertainty will be increased on the other parameters estimated (e.g. the total incidence) though this might reduce the overall error.

Our method removes the possible negative changes in incidence due to sampling variability when the number of sera tested and change of seroprevalence between two months are small. Its accuracy as a population measure will however depend on the representativeness of the sera included in the survey. Ideally sera that are obtained by random population sampling should be used rather than sera obtained from individuals bled for other purposes. However, use of residual aliquots of opportunistically available samples seems a good proxy in the case of an emerging infection such as pandemic influenza where there are high population attack rates that are largely age-dependent. More relevant is whether the change in the proportion with positive titers only reflects acquisition of antibody from infection. In the United Kingdom, vaccination with the A/H1N1 (2009) strain began in late November 2009 for individuals in clinical risk groups in whom the consequences of infection were shown to be more severe [17], and from January 2010 was recommended for all children under 5 years of age [18]. However, the impact of vaccination in our data set is likely to be small due to the low uptake rates [5] and has not been taken into account in this methodological paper. Another group where changes in seroprevalence may not reflect their own infection are young infants in whom the detection of H1N1 antibody in the first few months of life may result from pre-natal acquisition of maternal antibody rather than post-natal infection. For this reason we excluded infants under 1 year of age from our incidence analyses.

In addition to factors such as vaccination or antibody decay which may influence the change in seroprevalence, there are likely to be factors such as changes over time in the propensity to consult or changes in proportion of patients tested for infection as the pandemic evolves that will influence the shape of the incidence curve derived from clinical or laboratory-based surveillance data. Theoretically the shape of the incidence curve does not need to be defined though in practice this might be difficult to implement because of the large increase in parameter space needed to describe the numerous putative incidences. Incorporating such additional factors would require a more complex set of parameters to be estimated in order to derive sero-incidence. For this MCMC could be used with the likelihood function from Equation 2 to draw a sample from the parameter space and then the parameters can be evaluated from the sampled distribution.

Due to the necessary lag times in sample collection and testing, generation of seroincidence data will inevitably have a built-in delay. However, to be useful for policy makers, “real time” predictive models require more timely estimates of how incidence is changing, particularly in the early stages of the pandemic. This will require the use of clinical surveillance data derived from individuals accessing health care which can be available daily if required. While such data may accurately reflect the time course of the pandemic, it will underestimate the true incidence of infection and requires the use of a scaling factor for model parameterization reflecting an estimate of the consultation rate per infection, as described by Baguelin et al. [1]. Moreover, estimation of the infection rate using clinical surveillance data is difficult until the epidemic peaks when the depletion of susceptibles can then be relatively accurately calculated providing there is an independent measure of the basic reproductive ratio *R_0_*. The method presented in this paper provides an improved tool to estimate incidence earlier in an emerging epidemic, thus allowing earlier estimation of the scaling factor that needs to be applied to the clinical surveillance data used for model updating. As more serologic data become available, revised estimates of the scaling factor can then be produced using a Bayesian framework (the current estimate is used as prior and the new samples provide a new likelihood function).

Our estimated number of infections in the second wave were 20 to 40 fold higher than the number of clinical cases estimated by the HPA, based on ILI consultations in individuals with confirmed H1N1 (2009), scaled up by the estimated proportion of patients with ILI who seek health care [6]. The difference between the clinical case estimates and our infection estimates will reflect both the proportion of infections that are asymptomatic and the real proportion of patients with ILI seeking health care. The fold difference between infections and the HPA clinical case estimates was around 10 in the first wave [1], [2]. Since the symptomatic/asymptomatic ratio is unlikely to have changed between the first and second waves, these fold differences are consistent with the suggestion that there was a lower propensity to consult in the second than the first wave [19].

In summary, our likelihood-based estimation method provides a more accurate measure of incidence than achieved by comparing prevalence in samples grouped by time period. It allows the generation of a continuous curve that describes how incidence is changing over the course of the pandemic and removes the possibility of generating negative incidence estimates by sampling error. It also obviates the need for pre-pandemic samples to establish the antibody prevalence at baseline. The method has potential for further development to incorporate and estimate the effect of other variables influencing changes in antibody prevalence, such as vaccination or waning immunity after infection. It has general applicability to any sequential serologic data set obtained over a period of changing incidence.
